# Heterochromatin in the fungal plant pathogen, *Zymoseptoria tritici*: Control of transposable elements, genome plasticity and virulence

**DOI:** 10.3389/fgene.2022.1058741

**Published:** 2022-11-21

**Authors:** Callum J. Fraser, Simon K. Whitehall

**Affiliations:** Biosciences Institute, Newcastle University, Newcastle upon Tyne, United Kingdom

**Keywords:** heterochromatin, *Zymoseptoria tritici*, histone H3 lys 27 trimethylation, histone H3 lys 9 methylation, transposable element, fungal plant pathogen, genome plasticity

## Abstract

Heterochromatin is a repressive chromatin state that plays key roles in the functional organisation of eukaryotic genomes. In fungal plant pathogens, effector genes that are required for host colonization tend to be associated with heterochromatic regions of the genome that are enriched with transposable elements. It has been proposed that the heterochromatin environment silences effector genes in the absence of host and dynamic chromatin remodelling facilitates their expression during infection. Here we discuss this model in the context of the key wheat pathogen, *Zymoseptoria tritici.* We cover progress in understanding the deposition and recognition of heterochromatic histone post translational modifications in *Z. tritici* and the role that heterochromatin plays in control of genome plasticity and virulence.

## Introduction

### The wheat pathogen, *Zymoseptoria tritici*

*Zymoseptoria tritici* is responsible for *Septoria tritici* blotch (STB), a world-wide foliar disease of wheat that is characterised by the appearance of necrotic lesions and in some cases the death of the plant. Yields in an infected crop may be reduced by 30–50% if the disease is not properly controlled *via* the application of fungicides (Fones and Gurr, 2015). However, the increasing incidence of fungicide resistance means that *Z. tritici* is a major threat to wheat production (Torriani et al., 2015).

Infection is initiated when *Z. tritici* spores on the leaf surface germinate and hyphae invade plant tissue through stomata. This is followed by a slow, asymptomatic colonisation of the apoplastic space of the surrounding mesophyll. After approximately 10 days (depending on temperature), there is an abrupt switch from “stealth” infection to necrotrophic growth during which plant cells undergo programmed cell death. The resulting release of nutrients from the dying plant tissue facilitates a rapid increase in fungal biomass and the formation of pycnidia which contain pycnidiospores that are released to infect the leaves of neighbouring plants (Steinberg, 2015).

## Effector genes, repetitive elements and compartmentalised genomes

Plant colonizing fungi produce a repertoire of secreted effector proteins that modulate plant immunity and host cell physiology (Uhse and Djamei, 2018). The expression of effectors is tightly regulated so that they are produced at the appropriate stage and level during infection (Uhse and Djamei, 2018). Accordingly, *Z. tritici* undergoes several major gene expression reprogramming events during the infection of wheat leaves (Kellner et al., 2014; Rudd et al., 2015). These events include a large up-regulation of secreted proteins approximately 9 days after infection which likely underpins the shift to necrotrophic growth. After approximately 14 days there is a general reduction in the expression of secreted proteins in favour of cell wall and carbohydrate degrading enzymes (Rudd et al., 2015). However, the mechanisms by which these large scale transcriptional reprogramming events are regulated remains to be determined.

The distribution of effector genes in the genomes of filamentous plant pathogens is not random. Instead effector genes, and genes involved in the production of secondary metabolites (SM), tend to be located in clusters within, or close to, regions of the genome that are enriched in repetitive sequences such as transposable elements (TEs) (Dong et al., 2015). An association with TEs is significant because it is increasingly clear that these elements can be utilised by cells as sources of genetic variability that facilitate genome evolution (Colonna Romano and Fanti, 2022). Mobilization of TEs to new sites in the genome may influence the expression of adjacent genes and also their repetitive nature makes them potential substrates for recombination and thus drivers of genomic rearrangements. That TE/repeat-enriched genomic regions are rapidly evolving has given rise to the “two speed genome hypothesis” where specific compartments provide sources of genetic plasticity that drive adaptation to the host species (Dong et al., 2015; Seidl and Thomma, 2017; Fouche et al., 2018).

## TEs and genome plasticity in *Z. tritici*

There is growing evidence indicating that TEs have shaped the compartmentalisation of the *Z. tritici* genome. *Z. tritici* has 13 essential core chromosomes and up to 8 conditionally dispensable accessory chromosomes that are gene poor and especially enriched with TEs (Goodwin et al., 2011). Accessory chromosomes are frequently lost during vegetative growth and are prone to rearrangements in meiosis with the breakpoints frequently mapping close to TEs (Wittenberg et al., 2009; Goodwin et al., 2011; Croll et al., 2013; Möller et al., 2019). Core chromosomes from different isolates also exhibit significant structural polymorphism resulting from the insertion or deletion of clusters of TEs (Plissonneau et al., 2016; Plissonneau et al., 2018). The TE-associated chromosomal regions that are present or absent in a strain specific manner (which have been termed accessory, orphan or dispensable regions), are enriched for putative effector genes and account for substantial variations in gene content. Indeed, comparative analysis of the genomes of five *Z. tritici* isolates identified a core set of 9149 genes and a further 6600 strain-specific accessory genes (Plissonneau et al., 2018).

## TEs and heterochromatin domains

Host cells employ a variety of defence mechanisms that restrict the activity of TEs to tolerable levels (Goodier, 2016). TEs are often embedded in heterochromatin, a condensed form of chromatin that is typically refractory to transcription and other DNA-dependent processes (Allshire and Madhani, 2018; Marsano and Dimitri, 2022). A key property of heterochromatin is the potential to spread from nucleation sites to cover extended domains (Allshire and Madhani, 2018) and as a result, genes in the vicinity of TEs can be subject to heterochromatic silencing (Choi and Lee, 2020). Indeed, the expression of effector and SM genes is suppressed by heterochromatin in a variety of plant-colonizing fungi (Connolly et al., 2013; Chujo and Scott, 2014; Soyer et al., 2014; Studt et al., 2016). As discussed further below, heterochromatin has also been linked to the pathogenicity of *Z. tritici*; putative effector genes that are upregulated during the switch to necrotrophic growth are enriched in heterochromatic regions of the genome (Meile et al., 2018; Soyer et al., 2019) and loss of key heterochromatin regulators interferes with the ability of *Z. tritici* to infect wheat leaves (Möller et al., 2019) (Table 1).

**TABLE 1:** Summary of the virulence phenotypes of *Z. tritici* heterochromatin mutants.

Mutant	Protein/Function	Virulence phenotype^a^	References(s)
Δ*kmt1*	Histone H3 lysine 9 methyltransferase	Severely impaired/abolished. No necrosis or pycnidia during screening period	Möller et al., 2019; Fraser et al., 2022
Δ*kmt6*	Histone H3 lysine 27 methyltransferase	Mildly impaired. Some reduction in necrosis and pycnidia	Möller et al., 2019
Δ*kmt1* Δ*kmt6*	Histone H3 lysine 9 & 27 methyltransferases	Severely impaired/abolished No necrosis or pycnidia during screening period	Möller et al., 2019
Δ*cbx1*	Heterochromatin Protein 1 (HP1)	Moderately impaired. Reduced pycnidia	Fraser et al., 2022
Δ*cbx2*	H3K9me-binding chromodomain protein	No detectable impairment	Fraser et al., 2022

^a^ As determined by wheat infection assays.

## Heterochromatin histone post-translational modifications

Heterochromatin can be subdivided into two basic forms: constitutive and facultative (Liu et al., 2020). Constitutive heterochromatin is typically associated with highly repetitive regions such as telomeres and centromeres and often plays an important architectural role in chromosomes. Facultative heterochromatin is considered to be a more fluid subclass of heterochromatin which can be remodelled in response to appropriate internal or external signals. For example, in metazoans it is associated with developmentally regulated genes and similarly, facultative heterochromatin regulates the expression of SM genes in some filamentous fungi (Liu et al., 2020; Ridenour et al., 2020).

Heterochromatic regions are characterised by the presence of specific histone post-translational modifications (PTMs). Di- and tri-methylation of histone H3 on lysine 9 (H3K9me2/3) are hallmarks of constitutive heterochromatin while facultative heterochromatin in metazoans is classically marked by histone H3 that is trimethylated on lysine 27 (H3K27me3) (Liu et al., 2020). The genomic locations of heterochromatic PTMs in a *Z. tritici* reference strain have been mapped using ChIP-seq (Schotanus et al., 2015). H3K9me3 was found to cover around 22% of the genome but only a very small number of protein coding genes were found to be associated with this PTM. Instead H3K9me3 predominantly maps to TEs and to subtelomeric regions. H3K27me3 is also found associated with subtelomeric regions and TEs but, in comparison to H3K9me3, has a broader genomic distribution that encompasses specific genic regions. Furthermore, accessory chromosomes are particularly enriched for H3K27me3. In contrast to some other organisms, pericentomeric regions of *Z. tritici* chromosomes are not marked with H3K9me3. Indeed, *Z. tritici* centromeres are quite unusual, being relatively small and lacking extensive AT–rich tracts (Schotanus et al., 2015).

The *Z. tritici* enzymes responsible for catalysing these modifications are conserved SET [Su (var), Enhancer of zeste, Trithorax] domain lysine methyltransferases (KMTases) (Qian and Zhou, 2006). H3K9me2/3 is mediated by Kmt1 which is a homolog of human Suv39H1/2 (*Schizosaccharomyces pombe* Clr4, *Neurospora crassa* DIM5) and H3K27me3 by Kmt6 which is a homolog of *Drosophila* E(Z) and human EZH2/1 (Möller et al., 2019). The functions of H3K9me2/3 and H3K27me3 in *Z. tritici* have been investigated through the analysis of mutant strains lacking these KMTases. Deletion of *kmt1* results in a severe reduction of fitness *in vitro* and sensitivity to a range of abiotic stresses (osmotic, oxidative and genotoxic). Furthermore, Δ*kmt1* strains have severely reduced virulence in wheat infections assays indicating that H3K9me2/3 is important for growth *in planta*. In contrast, loss of *kmt6* (and thus H3K27me3) does not reduce growth *in vitro* and is associated with only modest reduction in virulence (Möller et al., 2019) (Table 1).

H3K27me3 and H3K9me2/3 modifications play opposing roles in the regulation of genome stability. As outlined above, the H3K27me3-enriched accessory chromosomes are highly unstable and are frequently lost during cell division. Deletion of *kmt6* decreases the frequency with which these chromosomes are lost indicating that H3K27me3 promotes their instability (Möller et al., 2019). Increased H3K27me3 is also correlated with an elevated frequency of accessory chromosome loss in *Fusarium fujikuroi* (Janevska et al., 2018). Analysis of *kmt6* mutants has also demonstrated that H3K27me3 promotes the rate of spontaneous mutations (Habig et al., 2021). In contrast, H3K9me2/3 functions to suppress spontaneous mutation rates and genomic instability. Deletion of *kmt1* is associated with increased accessory chromosome loss, de-repression of TEs and an elevated incidence of chromosomal rearrangements with break points that often map close to TE clusters (Möller et al., 2019; Habig et al., 2021). The high level of chromosomal instability observed in *kmt1* mutants is driven by the widespread re-localisation of H3K27me3 such that it invades regions previously occupied by H3K9me2/3. Accordingly, deletion of *kmt6* in a Δ*kmt1* background suppresses genomic instability (Möller et al., 2019).

In addition to TEs, a large set of protein encoding genes is upregulated in the absence of *kmt1*, which is consistent with H3K9me2/3 playing an important role in gene silencing. However, the majority of these genes are not directly associated with H3K9me2/3 and the changes in their expression may result, at least in part, from the re-distribution of H3K27me3 that occurs in a *kmt1* mutant background (Möller et al., 2019). The majority of H3K27me3-marked genes and TEs located on core chromosomes are not activated by its removal and in contrast to some other filamentous fungi, heterochromatic PTMs do not play a major role in the control of *Z. tritici* SM genes (Möller et al., 2019; Hassani et al., 2022). However, heterochromatin is implicated in the regulation of effector gene expression and thus the interaction of *Z. tritici* with its host. Analysis of putative effector genes revealed a significant association with genomic loci that are marked with H3K27me3 or overlapping H3K27me2 and H3K9me3 under axenic growth conditions (Soyer et al., 2019). The regulation of four of these effectors (*AvStb6*, *Avr3D1*, *QTL_5*, and *Mycgr3G76589*) has been analysed using fluorescent reporter genes (Meile et al., 2020). These loci are silenced under axenic conditions but expression is induced during host colonization. Furthermore, the increase in expression at the switch to necrotrophic growth coincides with a decrease in the associated levels of H3K27me3 at all four genes and all but one also had a reduction in H3K9me3 levels. This is consistent with the active remodelling of heterochromatic domains during infection. Moving these effector genes to an ectopic location results in loss of silencing under axenic conditions and aberrant *in planta* expression indicating that genomic environment is an important facet of their regulation (Meile et al., 2020).

## Histone H3K9 KMTase complexes

**FIGURE 1:**
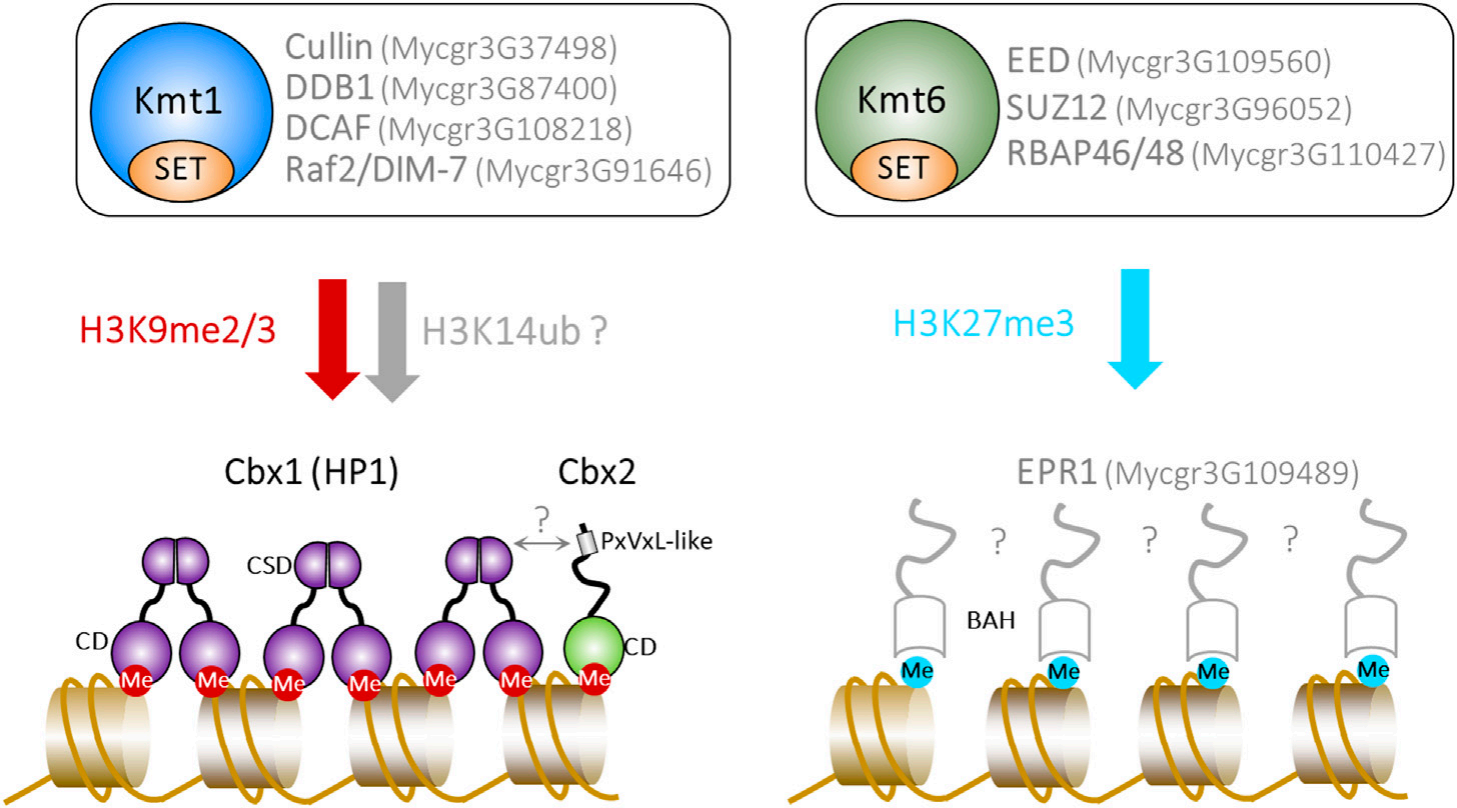
The deposition and recognition of heterochromatic histone PTMs in *Z. tritici.* The top panel shows the predicted composition of Kmt1 (histone H3K9) and Kmt6 (histone H3K27) KMTase complexes. The bottom panel summarises proteins known to be, or predicted to be, involved in the recognition of H3K9me2/3 and H3K27me3. Predicted proteins, activities and interactions are shown in grey. The Ensembl Fungi ID of genes encoding putative proteins is indicated.

*S. pombe* Clr4 and *N. crassa* DIM-5 H3K9 KMTases function in the context of Cul4 ubiquitin ligase complexes, which are called CLRC (Clr4 methyltransferase complex) and DCDC (DIM-5-7-9 CUL-4 DDB1), respectively (Hong et al., 2005; Horn et al., 2005; Jia et al., 2005; Li et al., 2005; Lewis et al., 2010a; Lewis et al., 2010b). Mutations in the genes encoding CLRC/DCDC subunits result in the loss of H3K9methylation in both organisms. This indicates that the integrity of these complexes is required for the catalytic activity of the KMTase and recent work has shown that CLRC ubiquitylates histone H3 on lysine 14 (H3K14ub), promoting H3K9me by Clr4 (Oya et al.). This H3K14ub-H3K9me “crosstalk” mechanism has not yet been investigated in *Z. tritici* but homologs of CLRC/DCDC subunits are present (Figure 1). Furthermore, Suv39H1 is associated with CUL4 in human cells (Yang et al., 2015) and knockdown of CUL4 reduces H3K9me levels (Higa et al., 2006). As such, a functional link between CUL4 mediated ubiquitylation and methylation of H3K9 may be broadly conserved.

## H3K9me2/3 readers in *Z. tritici*

Histone PTMs exert their function by acting as docking sites for proteins (termed “readers”) which modify chromatin structure and regulate DNA-dependent processes. The recognition of H3K9me2/3 PTMs is commonly mediated by the Heterochromatin Protein 1 (HP1) family of chromodomain (CD) containing proteins which are present in a variety of eukaryotic organisms including filamentous fungi (Kumar and Kono, 2020). HP1 proteins have a conserved architecture comprised of an N-terminal chromodomain (CD) and a C-terminal chromo-shadow domain (CSD) separated by a flexible hinge region. Recognition of H3K9me2/3 is achieved by the CD. Structural analyses have demonstrated that CDs fold in to a three-stranded β-sheet with an adjacent helix and that methyl-lysine co-ordination is achieved by a “cage” formed by the sidechains of three conserved aromatic residues (Jacobs and Khorasanizadeh, 2002; Nielsen et al., 2002). The CSD mediates homodimerization and provides a docking surface for the interaction of additional proteins with PxVxL and related motifs (Brasher et al., 2000; Cowieson et al., 2000; Huang et al., 2006). Cryo EM analysis has revealed that HP1 dimers are capable of bridging between adjacent nucleosomes (Machida et al., 2018) and further HP1 self-interactions are proposed to facilitate the formation of oligomeric structures which compact nucleosome arrays and provide a platform for the recruitment of additional chromatin regulatory complexes (Canzio et al., 2011; Kumar and Kono, 2020).

Many species have multiple HP1 paralogs (e.g. humans and mice have three, *Drosophila* has five and *S. pombe* has two) but filamentous fungi often have a single isoform. The sole HP1 ortholog in *Z. tritici,* Cbx1, binds to both H3K9me2 and me3 *in vitro* and is enriched at H3K9me-marked loci *in vivo* (Fraser et al., 2022). Transcriptomic analysis indicates that Kmt1 and Cbx1 regulate the expression of highly similar sets of protein encoding genes. Surprisingly, loss of *cbx1* does not result in a global increase in transcripts derived from TEs and unlike Δ*kmt1* mutants, Δ*cbx1* strains are not sensitive to abiotic stresses and exhibit only a moderate reduction in virulence in wheat infection assays (Fraser et al., 2022) (Table 1).

The different phenotypes associated with loss of the HP1 (Cbx1) and the H3K9 methyltransferase (Kmt1) are suggestive of additional H3K9me readers in *Z. tritici* and indeed a second CD domain protein (Cbx2) that binds to H3K9me has recently been identified (Fraser et al., 2022). In contrast to the conserved HP1 protein, Cbx1, homologs of Cbx2 are restricted to species in just a few fungal families (mainly the *Mycosphaerellaceae* and *Teratosphaeriaceae*). Cbx2 is unusual in that it contains two CDs located in the C-terminal region both of which have the conserved “aromatic cage” residues (Fraser et al., 2022). The remaining region lacks any other obvious protein domains and is predicted to be largely unstructured. Mutation of *cbx2* alone does not result in marked growth defects either *in planta* or *in vitro.* However, combining deletions in *cbx1* and *cbx2* mimics the sensitivity of Δ*kmt1* strains to abiotic stresses consistent with the downstream function of H3K9me PTMs being mediated by a combination of these CD proteins (Fraser et al., 2022). Furthermore, Cbx1 and Cbx2 play redundant roles in the silencing of some Kmt1-regulated genes. *In vitro* analysis of Cbx2 indicates that it shows a strong preference for tri-methylated H3K9 peptides. This is potentially significant as analysis of H3K9me2 and me3 marks in *S. pombe* have revealed that they demarcate functionally different types of heterochromatin that are associated with different levels of transcriptional silencing (Jih et al., 2017).

While the role of Cbx2 in the establishment and or maintenance of constitutive heterochromatin remains to be determined, it is interesting to note that it shares features with the *N. crassa* heterochromatin regulator CDP-2 (Honda et al., 2012). Both proteins have CDs located in their C-terminal regions with much of the remaining sequence predicted to be unstructured. Furthermore, alignment of Cbx2 and CDP-2 homologues has revealed a small block of homology in the N-terminal region. CDP-2 is a subunit of a HP1-containing histone deacetylase complex called HCHC which functions in parallel with a HP1-DNA methyltransferase complex (HP1-DIM2) to establish and maintain heterochromatin in *N. crassa*. The N-terminal region of CDP-2 has a PxVxL-like motif which is required for interaction with the CSD of *N. crassa* HP1 (Honda et al., 2012). Interestingly, *Z. tritici* Cbx2 has two sequences in its N-terminal region that conform to a proposed CSD binding motif Φx(V/P)x(L/M/V) (Huang et al., 2006). It will therefore be important to determine whether or not Cbx2 functions in a HDAC complex with the HP1 homolog Cbx1.

## H3K9me3 and DNA cytosine methylation

DNA cytosine methylation (5 mC) is an additional feature of constitutive heterochromatic loci in many organisms. The deposition of 5 mC has been well studied in *N. crassa* and is mediated by the DNA methyltransferase (DNMT) DIM-2 which is targeted to H3K9me3-marked genomic loci through a direct interaction with HP1 (Honda and Selker, 2008). While many *Z. tritici* isolates (including the commonly studied reference strain IPO323), lack functional copies of *dim2* and thus 5 mC, several Iranian *Z. tritici* isolates have been identified which have a functional *dim2* allele and high levels of 5 mC at TEs (Möller et al., 2021). Comparison of the genomic patterns of H3K9me3 and 5 mC revealed the co-localization of these modifications suggesting an interaction between Cbx1 (HP1) and Dim2 similar to that observed in *N. crassa*. In fungi, 5 mC is mainly associated with TEs and is involved in a genome defence mechanism called RIP (Repeat-Induced Point mutation) which is underpinned by the deamination of 5 mC to thymine leading to the accumulation of C to T transitions in repeated elements (Gladyshev, 2017). Accordingly, the presence of functional Dim2 increased mutation rates associated with *Z. tritici* TEs (Möller et al., 2021).

## Mechanisms of H3K27me3 deposition and recognition in *Z. tritici*

The deposition and recognition of H3K27me3 in metazoans is mediated by Polycomb repressive complexes 1 and 2 (PRC1 and PRC2), respectively. PRC2 type complexes contain a H3K27 KMTase, together with other core subunits: EED, SUZ12, and RBBP4/7 (Laugesen et al., 2019). A variety of other ‘accessory’ proteins are associated with mammalian PRC2 complexes at substoichiometric levels. The EZH2 KMTase is catalytically inactive in the absence of the other PRC2 subunits and structural studies have revealed that it undergoes a conformational change in the presence of SUZ12 and EED which results in the correct positioning of the methyl donor (*S*-adenosyl methionine) and the ε-amino group (Jiao and Liu, 2015). PRC1 functions as a reader of H3K27me3 and is thought to bring about transcriptional silencing by directing the compaction of chromatin and the monoubiquitination of histone H2A on lysine 119 (H2AK119Ub) (Piunti and Shilatifard, 2021). Mammals have a variety of PRC1 type complexes which vary in their subunit composition with binding to H3K27me3 being mediated by one of five chromobox (CBX) proteins which are orthologs of the *Drosophila* CD protein Polycomb (Pc) (Piunti and Shilatifard, 2021).

PRC2 subunits are generally conserved in fungi that mediate H3K27 trimethylation including *Z. tritici* (Ridenour et al., 2020). An exception is the *Cryptococcus neoformans* PRC2 complex which apparently lacks a SUZ12 equivalent (Dumesic et al., 2015). Functional analyses of the putative *Z. tritici* EED (Mycgr3G109560), SUZ12 (Mycgr3G96052) and RBAP46/48 (Mycgr3G110427) subunits are currently lacking. Nonetheless, it appears that *Z. tritici* possesses a PRC2 complex and that the fundamentals of H3K27me3 deposition are conserved with metazoans (Figure 1). In stark contrast, PRC1 subunits have not been identified in fungi and the mechanisms by which this PTM mediates its biological downstream effects are poorly understood. Despite H3K27me3 being present in a variety of fungal species, to date only one fungal CD protein- *Cryptococcus neoformans* Ccc1-has been identified that is capable of specifically binding to this PTM (Dumesic et al., 2015). Ccc1 is a component of the PRC2 complex and its loss causes the redistribution of H3K27me3 to regions occupied by H3K9me indicating that Ccc1 restrains PRC2 activity to correct regions of the genome (Dumesic et al., 2015). The degree to which this is a feature of fungal PRC2 complexes remains to be determined as orthologs of Ccc1 have not been identified in other species.

Analysis of a *Z. tritici* reference genome indicates that it encodes a relatively limited repertoire of CD proteins with the potential to recognise heterochromatic histone PTMs. While the HP1 proteins of some organisms recognise H3K27me3 (Turck et al., 2007; Yale et al., 2016), neither Cbx1 nor Cbx2 show any specificity for H3 peptide tails methylated on K27 *in vitro* (although an *in vivo* role cannot be completely discounted) (Fraser et al., 2022). A major role for any of the remaining hypothetical CD proteins (termed Cbx3-Cbx6) in the recognition of H3K27me3 is also unlikely as these proteins lack critical conserved caging aromatic amino acids and/or are encoded by genes on the accessory chromosomes. Some also exhibit similarity to retroviral CD-containing integrases suggesting they are remnants of retrotransposable elements (Fraser et al., 2022).

Recent studies have provided exciting insights into mechanisms of H3K27me3 recognition and silencing independent of PRC1. Using a forward genetic screen in *N. crassa*, Wiles and colleagues identified EPR-1, a protein with BAH (bromo-adjacent homology) and PHD (plant homeodomain) motifs that is required for transcriptional repression at H3K27me3 marked genes. EPR-1 associates with H3K27me3 marked loci in a manner which is dependent upon the BAH domain (Wiles et al., 2020). Furthermore, the BAH domain of the *Fusarium graminearum* EPR-1 orthlog, BP1 binds to methylated H3K27 peptides and loss of BP1 de-represses the expression of K27me3-regulated SM genes (Tang et al., 2021). EPR-1 homologs are present in a variety of eukaryotic lineages (Wiles et al., 2020) and indeed BAH-PHD domain proteins have also been linked to PRC2-mediated silencing in plants, which like fungi, lack PRC1 components (Li et al., 2018; Qian et al., 2018; Yang et al., 2018). The BAH domains of human BAHD1 and BAHCC1 have also been shown to function as H3K27me3 readers and BAHD1 is required for optimal silencing of H3K27me3 marked genes (Zhao et al., 2016; Fan et al., 2020; Fan et al., 2021; Xu et al., 2021). Thus BAH mediated recognition of H3K27me3 is conserved across fungi, plants and animals suggesting that BAH represents a more ancient class of Polycomb silencing (Wiles et al., 2020). Characterisation of a putative *Z. tritici* EPR-1 homolog (Mycgr3G109489) has not yet been reported but it will be important to determine its role in the stability of accessory chromosomes and the control of effector gene expression.

## Discussion

While it has been established that heterochromatic PTMs have an important influence on the genome evolution, effector gene expression and virulence of *Z. tritici* (Möller et al., 2019; Soyer et al., 2019; Meile et al., 2020; Habig et al., 2021), there are a number of questions surrounding their deposition, maintenance and function that remain to be resolved. Firstly, how are Kmt1 and Kmt6 KMTases targeted to specific regions of the genome? Little is known about the recruitment of Ezh2/Kmt6 type enzymes in fungi and even in relatively well-studied mammalian systems, understanding of PRC2 recruitment is incomplete (Guo et al., 2021). In *S*. *pombe*, genomic targeting of Clr4 (H3K9me KMTase) is linked to the RNAi machinery (Martienssen and Moazed, 2015) but no functional link between RNAi and heterochromatin has been established in *Z. tritici*. Secondly, what are the mechanisms by which constitutive heterochromatin domains are spread and maintained through successive cell divisions in filamentous fungi such as *Z. tritici*? Unlike their counterparts in metazoans and fission yeast, the Suvar39/Clr4 KMTases of filamentous fungi do not have a recognisable CD in the N-terminal region. This is significant because for human Suv39H1 and *S. pombe* Clr4 the interaction of the CD with H3K9me2/3 is required for the spreading and maintenance of heterochromatic domains (Zhang et al., 2008; Muller et al., 2016). Thirdly, do all of the phenotypes associated with deletion of *kmt1* and *kmt6* result from loss of histone modifications? Mammalian Suv39H1 and Ezh2 KMTases both have non-histone substrates (Rodriguez-Paredes and Lyko, 2019; Weirich et al., 2021) and the possibility that this is the case for *Z. tritici* Kmt1 and Kmt6 needs to be considered. Finally, to what extent does the remodelling of heterochromatin during infection regulate the expression of effector genes (Soyer et al., 2019; Meile et al., 2020) and what are the environmental cues and signalling pathways that control this process? Here it is worth noting that the activity of eukaryotic TEs is often up-regulated in response to environmental stresses (Pappalardo et al., 2021; Ito, 2022) and the colonization of host tissue is a stress-inducing process (Sanchez-Vallet et al., 2018). Indeed, the upregulation of a large number of *Z. tritici* TE families coincides with the development of symptoms on wheat leaves (Fouche et al., 2020). Therefore, stress-responsive TEs could be an important facet of the controls that shape the chromatin environment of effector genes and thus the interaction of *Z. tritici* with its host.
